# New molecular targets in Hodgkin and Reed-Sternberg cells

**DOI:** 10.3389/fimmu.2023.1155468

**Published:** 2023-05-15

**Authors:** Hummaira Sadaf, Maciej Ambroziak, Robert Binkowski, Jakkapong Kluebsoongnoen, Ewa Paszkiewicz-Kozik, Jaroslaw Steciuk, Sergiusz Markowicz, Jan Walewski, Elzbieta Sarnowska, Tomasz Jacek Sarnowski, Ryszard Konopinski

**Affiliations:** ^1^ Department of Experimental Immunotherapy, Maria Sklodowska-Curie National Research Institute of Oncology, Warsaw, Poland; ^2^ Department of Biotechnology, Sardar Bahadur Khan Womens’ University, Balochistan, Pakistan; ^3^ Institute of Biochemistry and Biophysics Polish Academy of Sciences, Warsaw, Poland; ^4^ Department of Lymphoid Malignancies, Maria Sklodowska-Curie National Research Institute of Oncology, Warsaw, Poland

**Keywords:** Hodgkin lymphoma, Reed-Sternberg cells, chromatin remodeling, PD-1, PD-L1, CD30, ncRNA

## Abstract

Recent discoveries shed light on molecular mechanisms responsible for classical Hodgkin lymphoma (HL) development and progression, along with features of Hodgkin – Reed and Sternberg cells (HRS). Here, we summarize current knowledge on characteristic molecular alterations in HL, as well as existing targeted therapies and potential novel treatments for this disease. We discuss the importance of cluster of differentiation molecule 30 (CD30) and the programmed cell death-1 protein (PD-1) and ligands (PD-L1/2), and other molecules involved in immune modulation in HL. We highlight emerging evidence indicating that the altered function of SWI/SNF-type chromatin remodeling complexes, PRC2, and other epigenetic modifiers, contribute to variations in chromatin status, which are typical for HL. We postulate that despite of the existence of plentiful molecular data, the understanding of HL development remains incomplete. We therefore propose research directions involving analysis of reverse signaling in the PD-1/PD-L1 mechanism, chromatin remodeling, and epigenetics-related alterations, in order to identify HL features at the molecular level. Such attempts may lead to the identification of new molecular targets, and thus will likely substantially contribute to the future development of more effective targeted therapies.

## Highlights

- HL is characterized by CD30 and PD-L1 overexpression, as well as various epigenetic alterations;
- Further epigenetic study may broaden the knowledge of regulatory processes that are impaired in HL;
- CD30 and reverse signaling of PD-L1 partially overlap and are interdependent, although the exact molecular mechanism of their interaction remains elusive. Targeting both CD30 and PD-L1 likely represents a more robust strategy to treat HL than existing individual approaches;
- The precise influence of CD30 and reverse signaling of PD-L1 pathways on chromatin remodeling machinery and epigenetic changes in HL needs to be elucidated;
- The use of compounds targeting epigenetic machinery aberrant in HL represents an attractive future direction in designing new HL treatments.

## Introduction

Hodgkin Lymphoma (HL) is rare B-cell lymphoid malignancy with incidence of 2-3 new cases per 100,000 individuals annually. According to NCI NIH database it is estimated that in 2022 the 8,540 new HL cases will be diagnosed and 920 patients will die (https://seer.cancer.gov/statfacts/html/hodg.html). HL has a bimodal distribution - it mostly affects young adults, as well as those aged over 50. It is characterized by high cure rates. However, around 10–15% of early stage, and up to 30% patients of advanced stages, experience progressive or relapsed disease after chemotherapy or combined modality treatment. Salvage therapies followed by autologous stem cell transplantation (ASCT) remain the standard therapeutic strategy in the relapse setting, providing long term survival for about 40% of patients. Given that the average age of HL patients is mid-30s, survivors are expected to be economically active in society. Therefore, developing novel therapies, increasing effectiveness of conventional induction strategies, or creating new treatment options for relapse and refractory HL are economically and societally attractive aims.

The main pathological feature of HL is the presence of Hodgkin and Reed-Sternberg cells (HRS). These usually represent a few percent of tumor tissue mass, and are surrounded by an abundant polyclonal immune background. The cell type was first observed by Carl Sternberg in 1898, and Dorothy Reed Mendenhall in 1902, in microscope images of lymph nodes (1). HRS cells are characterized by large nuclei with a peculiar morphology. Frequently, the cells are bi- or multi-nucleated (2). HRS cells display a characteristic immunophenotype: CD30+, CD15+, CD3−, CD20−, and CD45− (3). They are surrounded by a variety of polyclonal T helper cells, NK cells, TAMs (Tumor Associated Macrophages), DCs (Dendritic Cells), mast cells, eosinophils, stromal cells, plasma cells, fibroblasts and other cells (4). HRS cells produce cytokines, including: interleukins (IL-5, IL-6, IL-7, IL-9, IL-10), granulocyte-macrophage colony-stimulating factor (GM-CSF), transforming growth factor β (TGF-β) and lymphotoxin α (5), which modulate the tumor microenvironment. All intercellular interactions and the molecular pathways interplay, appearing to form hubs which aid survival and growth of malignant HRS cells (6).

Recent discoveries in HRS cells suggest that CD30 or programmed cell death protein-1 receptor and ligands (PD-1/-PD-L1/2) are important pathways for HL development. As such, they have become targets for novel therapeutic strategies (7, 8). Moreover, the precise changes in chromatin state, resulting from altered function of chromatin remodeling complexes or other epigenetic modifiers, additionally emerge as a potential novel direction for HL treatment research. However, these have yet to be assessed in clinical trials.

Here, we provide an overview of current knowledge of HL molecular features, targeted therapies and molecular mechanisms. We propose new directions of study aimed at better understanding HL etiology and growth mechanisms. We highlight the significance of reverse signaling in the PD-1/PD-L1 pathway, the role of CD30 signaling, and interplay between these pathways, chromatin-related machinery and epigenetic changes. Collectively, this understanding forms a valuable basis for personalized HL treatment.

## Main molecular signaling pathways in HL

The survival and proliferation of HRS cells depend on various molecular aberrations, among them signaling involving CD30, and the PD-1/PD-L1,2 pathway seem to be very important.

### The CD30 as a hallmark in HL

CD30 signaling is an important pathway for survival and proliferation of HRS cells. CD30 forms a 120 kDa type I transmembrane glycoprotein, with both extracellular and intracellular domains. CD30 belongs to the tumor necrosis factor receptor superfamily (TNFRSF8) (9), and the cytoplasmic end of CD30 protein contains TNF receptor associated factor binding sequences (10) that can activate the pathway of the NFκB transcription factor (11). The human *CD30* gene is located on chromosome 1p36 (12). The protein is exclusively localized on a restricted number of B and T cells in healthy individuals (13). CD30 is also expressed in various malignant cell types, for instance on HRS cells, for which they are a molecular marker (14). In diseased patients, CD30 protein has also been observed in soluble form, for example in serum of HL patients (15).

The CD30 signaling pathway begins with stimulation *via* its extracellular domain. After stimulation, receptor trimerization is promoted, enabling signal transduction. The intracellular segment of CD30 then connects with TNFR-associated factors (16). Three TNFR-associated factors – TRAF1, TRAF2 and TRAF5 – activate two internal pathways. The first of these pathways is mediated by NFκB (17). Alternatively, the second pathway is associated with MAPK, and extracellular signal-regulated kinase (ERK1 and ERK2) is activated (18).

CD30 is capable of interactions with various molecules, and therefore plays an important role in numerous biological processes in different cell types. For instance, the CD30 molecule may inhibit expression of Fas-L (Fas ligand), granzyme B and perforin, as well as leading to deranged cytotoxicity. CD30 suppresses *c-myc* function, and strongly induces the CCR7 molecule, causing its induction and up-regulation. This suggests that CD30 signaling may be involved in homing of lymphocytes to lymph nodes. In addition, CD30 involvement in up-regulation of pro-apoptotic molecules strongly indicates its potential role in the apoptosis-promoting process (19). On the other hand, increased activity of TNFR-associated factor 1 and cellular inhibitor of apoptosis 2 protects cells from apoptosis. This observation suggests that CD30 may have a dual function, depending on cell type (20). Some data indicates that overexpression of CD30 in HL is linked to ligand-independent stimulation of NFκB, promoting tumor cell survival. However in the same reports, it was found that constitutive activation of NFκB in HL cells is not related to CD30 overexpression. Moreover, upregulation of CD30 in HRS cells through activation of the MAPK/ERK pathway leads to expression of the AP-1 transcription factor family member JunB (21).

The potential therapeutic use of CD30 as a molecular target has been studied widely. Gaspal et al. studied the effect of CD30 signaling in autoimmune disease prevention. Their results suggested that CD30 may be crucial for CD4 memory responses and development of autoimmune response (22). SGN-35, or brentuxiab vedotin, a humanized antibody targeting CD30 linked with monomethyl-auristatin E, has been widely used in clinical practice for over a decade (23, 24). The effect of this antibody on HRS cells occurs *via* internalization of the antibody in the CD30-receptor endocytosis process, resulting in death of the tumor cell (25).

CD30 is a valuable target for Chimeric Antigen Receptor T cell (CAR-T) therapy. In clinical trials, CD30 CAR-T therapy was shown to be safe and effective in heavily pre-treated patients with relapsed or refractory HL (26).

### Programmed cell death protein-1 receptor and ligands

Immune system activity is moderated by immune checkpoints that integrate co-stimulatory and inhibitory signals (27). Programmed cell death protein-1 (PD-1), also known as CD279, (encoded by *PDCD1* gene located on chromosome 2q37.3) is the most studied inhibitory checkpoint (28, 29). The Honjo group discovered CD279, an inhibitory receptor identical to PD-1, in 1992 (30). PD-1 is a trans-membrane receptor type I protein, which is usually expressed on cell surfaces of monocytes, dendritic cells, natural killer, T and B cells.

PD-1 has two naturally occurring ligands: PD-L1 (*CD274* or B7-H1) and PD-L2 (*CD273* or B7-DC). The genes encoding the two ligands of PD-1, PD-L1 and PD-L2, are located on chromosome 9p24.1, only 42 kilobases apart (31, 32). PD-1 and its PD-L1 ligand are essential for self-tolerance and preventing autoimmunity in healthy people. Tonsils, placenta, dendritic cells, monocytes in the liver and lungs are the only normal tissues that express PD-L1 (33). A unique feature of HRS cells is increased expression of PD-L1 and PD-L2 (34, 35).

The immune checkpoint blockade (ICB) of PD-1 receptor, which restores effector T-cell functions, has led to development of novel therapeutic strategies in many tumor types, including HL (36).

**Figure 1:**
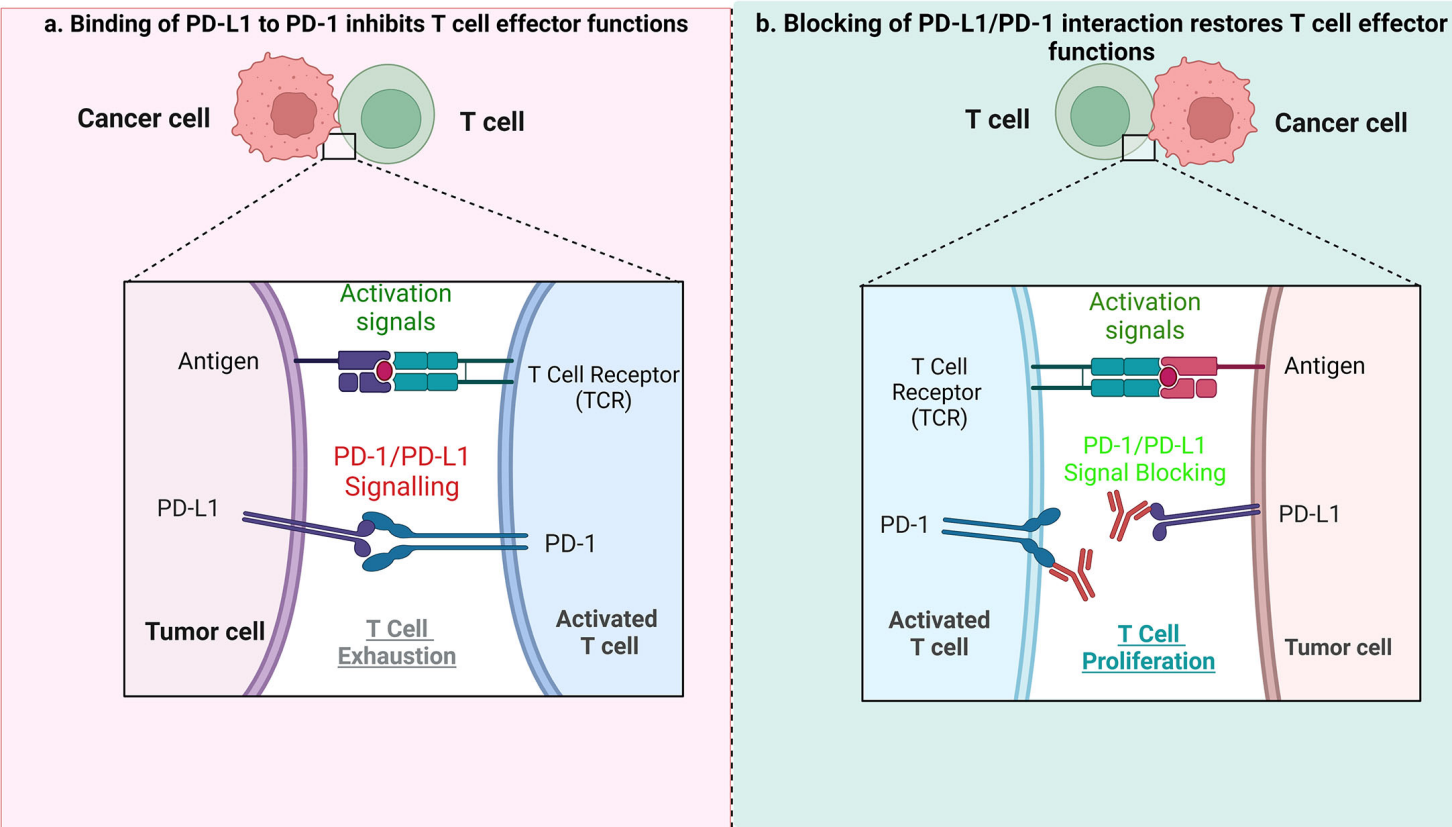
The role of PD1/PD-L1 axis and its inhibition in tumor treatment. **(A)** When PD-L1 expressed by tumor cells interacts with PD-1 on T cells, it results in suppression of effector T-cell function, which in turn limits T-cell proliferation, ultimately leading to the immune suppressive microenvironment. **(B)** When the PD1/PD-L1 axis is blocked, it causes T-cell proliferation, and restoration of their antitumor function.

Indeed, in HL, blockading the PD-1/PD-L1 axis is beneficial during patient treatment (37–39). PD-L1 expression can be increased using cytokines, including interferon γ (IFN-γ) and interleukin-4 in inflammatory conditions or during chronic inflammation. The PD-1/PD-L1 axis plays an important role in signal transduction. Although PD-1 is not expressed in anergic or inactive T cells, it can be induced after activation of T cells. Essentially, after binding with PD-L1/2 ligands, PD-1 transduces inhibitory signals to the T-cell receptor (TCR) pathway. The PD-1/PD-L1/2 interaction is responsible for inhibition of the T-cell receptor, mediation of lymphoid maturation and activation, as well as downregulation of the immune response. Blocking the interaction of either PD-1 or PD-L1 enhances T cell responses to cancer. Immune evasion – the avoidance of antitumor immune response - is achieved by suppressing PD-1 function. PD-1 expressed on tumor infiltrating T lymphocytes (TILs) interacts with PD-L1 or PD-L2 expressed on tumor cells (40, 41), leading to exhaustion of T cells. ICB treatment allows T cells to escape the inhibitory effects of tumor cells when the PD-1/PD-L1 pathway is blocked, and the anti-tumor immune response mediated by T cells is therefore restored (**Figure 1**).

Previous studies have indicated that patients with a variety of tumors, including relapsed/resistant lymphomas, have benefited from immunotherapy based on ICB. Anti-PD-1 antibodies such as nivolumab and pembrolizumab, that prevent PD-1 binding to its ligands, have been shown to significantly improve T cell immune response (proliferation and production of cytokines) as well as raising the response rate in patients with relapse/refractory HL (42, 43). The ICB treatment seems to be very effective in HL, likely due to a high expression of PD-L1 and/or PD-L2, a hallmark of HRS cells. These ligands are expressed mainly by HRS cells, as a consequence of increased expression from 9p24.1 *loci*, as well as by nonmalignant microenvironmental leukocytes (44). Several different mechanisms responsible for high level of PD-L1 expression in tumor cells have been reported. In HRS cells, chromosomal changes of 9p24.1 such as copy number gains, amplifications or translocations, may lead to increased PD-L1/2 gene expression. Such chromosomal aberrations are common in HL, with gains of 2p (most frequently, >50%), 12q, 17p, 9p, 16p, 17q and 20q and losses of 13q observed (45). A previous study indicated different percentages of PD-L1 and PD-L2 gene alterations, such as: polysomy - 5%; copy gain - 56%; amplification - 36%, respectively (46).

The chromosome region where *loci* encoding PD-L1/2 are located also contains the gene for Janus kinase 2 (JAK2), situated nearly 322 kilobases upstream from PD-L1. Amplification of the entire region increases the *cis-*influence of the modulation of JAK2 on PD-L1 promoter activity. It consequently enhances JAK2/STAT signaling in HRS cells, leading to both activation and, eventually, suppression (47, 48). Moreover, the JAK/STAT signaling pathway triggers expression of inflammatory cytokines, such as IL-10 in lymphoma cells. The activation of this cascade in HL enhances the proliferation and survival of tumor cells (49). Blocking this pathway could therefore reduce HRS cell proliferation by induction of apoptosis (50).

Considering the role of Epstein-Barr virus (EBV) in HL pathogenesis, infection with EBV is one possible inducer of PD-L1 and PD-L2 expression in this condition (51). EBV promotes AP1 and JAK/STAT signaling, increasing PD-L1 expression *via* an AP-1–dependent enhancer, through actions of the EBV-encoded latent membrane protein-1 (LMP1), regardless of 9p24.1 copy number amplification (52). These mechanisms may be particularly relevant in Western countries, where EBV is present in HRS cells in almost 40% of patients with HL (53).

Another mechanism of PD-L1 overexpression in HRS cells depends on activity of the AP-1 transcription factor. The *CD274* gene, which encodes PD-L1, has an AP-1 dependent enhancer that regulates PD-L1 expression in HRS cells. The AP-1 signaling components JunB/c-Jun persistently interact with this enhancer (54). Furthermore, another transcription factor, NFκB p50-RelA, undergoes constitutive activation in HRS cells. The cells subsequently exhibit increased levels of proliferation, dependent on NFκB activation. This transcription factor therefore possesses the ability to independently reduce stress-induced apoptosis in HRS cells (55). In general, NFκB is a well-known activator of PD-L1 expression in a broad spectrum of tumor and non-tumor cells (56).

The NPM/ALK tyrosine kinase also induces PD-L1 expression in HRS cells. NPM/ALK activates STAT3, which then functions as a transcriptional activator of the *CD274* gene, increasing its expression (57). Taking all of these examples together, the overexpression of PD-L1 and/or PD-L2 is a hallmark of HRS cells, although the precise mechanism of their overexpression may vary.

The main role of PD-L1 is as a ligand for the PD-1 receptor, therefore blockading the anti-tumor T cell immune response. Despite this, in HRS cells, reverse signaling involving the PD-L1 has been observed. This phenomenon is based on the function of PD-L1 as signal transducer upon PD-1 binding. In HRS cells, both membrane and soluble PD-1 may induce PD-L1 reverse signaling (given that in HL patients, high levels of soluble PD-1 have been observed) (58). Reverse signaling of PD-L1 plays an important role in terms of growth, proliferation and metabolism of HL. According to previous studies, reverse signaling of PD-L1 links to the MAPK cascade. An inverse relationship is reported between survival (proliferation) of the tumor cell and the MAPK cascade, in terms of phosphorylation of P38 and ERK–MAPK. Therefore, decreased phosphorylation of P38 leads to signals for survival and growth of the cell. Conversely, an increase in phospho-MAPK level would lead to apoptosis. It has also been demonstrated that blocking of reverse signaling through anti-PD-L1 antibodies may serve as an additional therapeutic strategy for HL (59). A previous study revealed the involvement of PD-L1 in regulation of cell proliferation, invasion and tumor growth. In the mouse lung cancer model cell line, the function of PD-L1 as a tumor-promoting factor was regulated by PI3K/Akt, Erk, and β-catenin, eventually targeting the WIP signaling cascade (60). To summarize, although existence of PD-L1 reverse signaling in HRS cells is already known, precisely which genes are directly regulated by, and the molecular pathways that may be connected to, the PD-L1 reverse cascade remain elusive. Deciphering this phenomenon may lead to a better understanding of the importance of PD-L1 in HL etiology, interplay with other immune cells in microenvironment, and serve as a basis for the development of novel treatment strategies.

**Figure 2:**
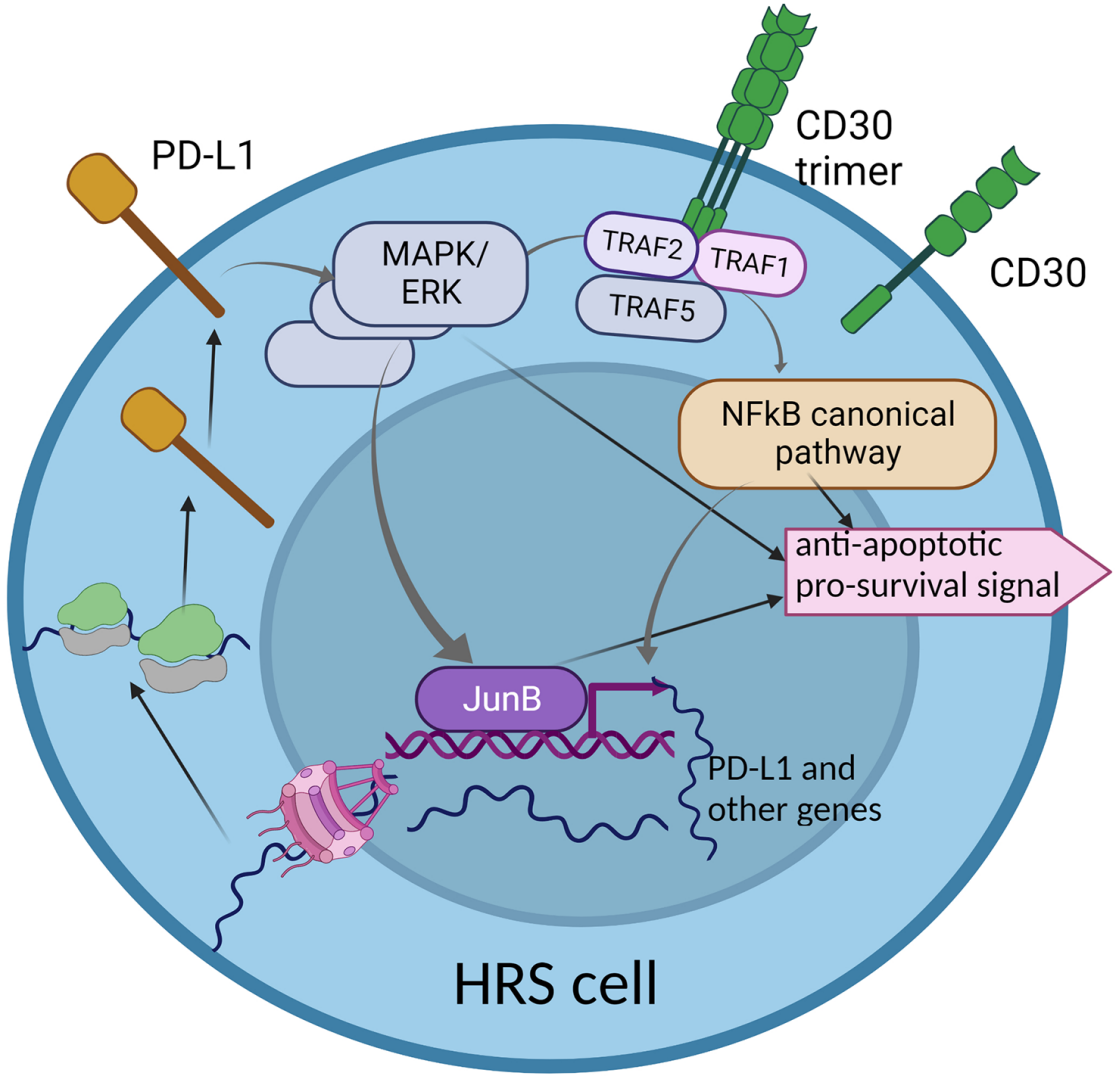
The interplay between two key molecular pathways: CD30 and PD-L1 reverse signaling. The CD30 signaling pathway partially overlaps with PD-L1 reverse signaling, through the MAPK/ERK cascade. This may suggest common functionality in activation of some genes that are important for HRS cell survival and proliferation. The hyper-activation of the CD30 pathway may enhance PD-L1 reverse signaling, which may be a characteristic phenomenon in HRS cells.

To conclude, CD30 and PD-L1 are two important molecules which are overexpressed in HRS cells. Both activate pathways which partially overlap and interplay, but still, little is known about their directed interdependencies and how the overexpression of CD30 may influence the expression of PD-L1 and *vice versa.* PD-L1 reverse signaling may lead to the activation of some genes, but whether the CD30-encoding gene is the target remains elusive. Therefore, understanding to what extent the CD30 and PD-L1 pathways overlap is vital, as combined therapy against both molecules may be more effective in refractory and relapse HL (**Figure 2**).

Activation of molecular pathways leads to particular genetic responses that are controlled by transcription factors, a broad spectrum of chromatin remodeling machineries, and other epigenetic modifiers. The over-activation of some pathways influences gene expression, and aberration in chromatin control machineries may lead to overexpression of various signaling molecules. Therefore, deep understanding of chromatin- and epigenetic-related mechanisms, and their respective roles in the control of altered signaling pathways in HL, may provide new insights into HRS cell function and HL development.

### Other molecules involved in immune modulation in HL

LAG-3 (Lymphocyte-Activation Gene 3) was found to be expressed in the HL microenvironment. Currently, inhibitors of LAG-3 molecule are in clinical trials. The recent data demonstrated that HL resistant for anti-PD1 immunotherapy was depleted in CD8+ T lymphocytes in tumor microenvironment and overexpressed the LAG-3 on CD4+ T lymphocytes. The LAG-3 molecule expression was found on a majority of TILs in pediatric HL (61). The safety and efficacy of MK-4280 (favezelimab), a humanized IgG4 LAG-3 inhibitor, in combination with pembrolizumab in relapse/refractory HL to anti-PD-1 therapy, was evaluated in clinical trials (**Table 1**) (61). Another molecule which may be important in relapse/refractory HL treatment is TIGIT (T cell Ig and ITIM domains). TIGIT is known as immune checkpoint receptor known to negatively regulate lymphocyte T functions. TIGIT is highly co-expressed with PD-1 molecule on both CD8+ and CD4+ T lymphocytes in cancers. Blockade of TIGIT using MK-7684 (vibostolimab) has strong antitumor activity in multiple pre-clinical tumor models (**Table 1**) (62). A multicohort, phase II clinical trial was designed to evaluate the safety and efficacy of vibostolimab in combination with pembrolizumab in patients with relapse/refractory HL (63). CD25 molecule is another potential target for immunotherapy in relapse/refractory HL. CD25 is highly expressed in effector T lymphocytes, Tregs and in non-immune cells (64). Camidanlumab tesirine the anti-CD25 antibody drug conjugate has been evaluated in clinical trial with promising results (**Table 1**) (65).

**Table 1:** Clinical trials related to HL.

Clinical trial number	Study title	Used treatment
NCT02665650	A Phase 1b Dose Escalation Study to Assess the Safety of AFM13 in Combination With Pembrolizumab in Patients With Relapsed or Refractory Classical Hodgkin Lymphoma (KEYNOTE- 206)	anti PD-1 mAb, CD30/CD16A-bispecific Ab
NCT05355051	A Phase II Study of the Combination of Azacitidine and Pembrolizumab for Patients Relapsed/Refractory Hodgkin’s Lymphoma	Anti PD-1 mAb, DNA methylation inhibitor
NCT05508867	A Study of Coformulated Favezelimab/Pembrolizumab (MK-4280A) Versus Physician’s Choice Chemotherapy in PD-(L)1-refractory, Relapsed or Refractory Classical Hodgkin Lymphoma (MK-4280A-008)	Anti LAG-3 mAb/anti PD-1 mAb
NCT04788043	Study of Magrolimab and Pembrolizumab in Relapsed or Refractory Classic Hodgkin Lymphoma	Anti CD47 mAb. Anti PD-1 mAb
NCT03471351	Safety and Efficacy Study of Tenalisib (RP6530) in Combination With Pembrolizumab in Relapsed or Refractory cHL	PI3K δ/γ inhibitor, anti PD-1 mAb
NCT03150329	Pembrolizumab and Vorinostat in Treating Patients With Relapsed or Refractory Diffuse Large B-Cell Lymphoma, Follicular Lymphoma, or Hodgkin Lymphoma	Anti PD-1 mAb, HDAC inhibitor
NCT05005442	A Study of Pembrolizumab/Vibostolimab (MK-7684A) in Relapsed/Refractory Hematological Malignancies (MK-7684A-004, KEYVIBE-004)	Anti TIGIT mAb/anti PD-1 mAb
NCT02432235	Study of ADCT-301 in Patients With Relapsed or Refractory Hodgkin and Non-Hodgkin Lymphoma	Anti – CD25 conjugated mAb
NCT00132028	Vorinostat in Treating Patients With Relapsed or Refractory Advanced Hodgkin’s Lymphoma	HDAC inhibitor
NCT00358982	Study of MGCD0103 (MG-0103) in Patients With Relapsed or Refractory Hodgkin’s Lymphoma	HDAC inhibitor
NCT00742027	Phase II Study of Oral Panobinostat in Adult Participants With Relapsed/Refractory Classical Hodgkin’s Lymphoma	HDAC inhibitor
NCT03603951	A Phase 1 Study of SHR2554 in Subjects With Relapsed or Refractory Mature Lymphoid Neoplasms	EZH2 inhibitor
NCT00866333	A Phase 2 Multi-Center Study of Entinostat (SNDX-275) in Patient With Relapsed or Refractory Hodgkin’s Lymphoma (ENGAGE-501)	HDAC inhibitor
NCT02961101	Anti-PD-1 Antibody Alone or in Combination With Decitabine/Chemotherapy in Relapsed or Refractory Malignancies	Anti PD-1 mAb, DNMT inhibitor
NCT03250962	SHR-1210 Alone or in Combination With Decitabine in Relapsed or Refractory Hodgkin Lymphoma	Anti PD-1 mAb, DNMT inhibitor

## Aberrant epigenetic mechanisms in HL

Among various alterations identified in malignant cells, genetic (e.g. mutations) and epigenetic changes play profound roles in cancer development and progression. Epigenetic mechanisms encompass methylation of cytosine residues in DNA, post-translational modification of histone proteins, nucleosome remodeling executed by multi-protein complexes, and actions of regulatory non-coding RNAs. Epigenetic regulatory mechanisms usually influence gene expression at the level of transcription, however they may also affect other steps of gene expression, such as translation. Given its broad regulatory effect on various processes, epigenetics was recently classified as an important hallmark of carcinogenesis. As various epigenetic alterations were reported recently in HL cells (66, 67), it is highly likely that systematic deciphering of the role of these non-mutational changes on the genetic sequence may improve understanding of the molecular mechanisms of the development, progression and aggressiveness of this cancer type.

### DNA methylation

Methylation of cytosines on CpG islands in DNA is one of the most common epigenetic modifications of regulatory regions in gene *loci*. This modification is introduced by DNA methyltransferases (DNMTs), which transfer the methyl group from the S-adenosyl-L-methionine (SAM) universal donor to the cytosines present in DNA. Therefore, DNA methyltransferases are classified as writers of epigenetic modifications. DNA methylation plays an important role in the control of gene expression at the transcriptional level. In tumorigenesis, CpG islands of the tumor suppressor genes are methylated, frequently resulting in altered chromatin structure, or in direct repression of gene expression. Hyperactivity of DNMTs and alterations in DNA methylation patterns are closely associated with various cancer types. It has been demonstrated that the HL cells exhibit downregulation/silencing of some characteristic genes including *CD19, CD20, CD79B, SYK, PU.1, BOB.1/OBF.1, BCMA*, and *LCK* (68). The silencing of these genes could be reversed by the introduction of 5-aza-deoxycytidine (5-aza-dC), suggesting that DNA hypermethylation may be involved in the control of their expression. Subsequent analysis indicated that DNA methylation is directly involved in the control of some of these genes, while in case of others, the expression could be dependent on additional genetic encoding, i.e. some master transcription factors (69–73). Array-based genome-wide study of DNA methylation in HL cell lines (74) led to the identification of hypermethylation on 383 CpG islands, corresponding to 329 genes which were commonly associated with characteristic changes for germinal center derived B-cell lymphoma (gcdBCL). These genes were mostly involved in development and morphogenesis, canonical Wnt receptor signaling regulation, and adenylate cyclase activity control. In contrast, 209 genes (247 CpGs) found to be specifically hypermethylated in HL cells were involved in positive regulation of B-cell activation and T-cell differentiation. It is noteworthy that *de novo* hypermethylation in HL resulted in the gene silencing. Unexpectedly, the majority (about 62%) of specifically silenced genes identified as hypermethylated in HL contained low CpG density in their promoter regions according to the current classification (75), suggesting the existence of an additional, and as yet unrecognized, epigenetic mechanism. The DNA methylation study additionally indicated that HL cells are rather characterized by DNA hypermethylation, as only a very small number of gene *loci* were specifically hypomethylated in HL (74). DNA methylation on promoter regions of *CD10, CD19*, and *LCK* genes was indicated as one of the factors distinguishing HL and lymphocyte predominant Hodgkin’s lymphoma (LPHL) at the molecular level, thus may be the reason for the appearance of characteristic features of Reed-Sternberg cells (76). Recent studies of DNA methylation indicated *HOXA5, MMP9, EPHA7* and *DAPK1* genes as markers enabling distinction between HL, mediastinal gray zone lymphoma, and primary mediastinal large B-cell lymphoma, with very high accuracy (77). Studies of DNA methylation in HL cells have therefore revealed several possibilities for drug repurposing studies, using DNMT inhibitors in HL treatment (and additionally in combined therapy) where they are already used for other cancer types (78, 79). DNMTs inhibitors, such as 5-azacytidine, have been shown to be effective in treatment of myelodysplastic syndromes (MDS) (80–82) and their use is becoming the standard of patients’ care within this group of cancers. In one case study, application of 5-azacytidine to a patient with relapsed HL resulted in a reduction of both, the tumor size and the metabolic activity of the disease (83). It is also hypothesized that hypomethylating agents such as 5-azacytidine might have an immune priming effect and can enhance the efficacy of immune checkpoint inhibitors, as patients previously treated with 5-azacytidine showed unprecedentedly increased response to subsequent administration of pembrolizumab or nivolumab (84). A couple of clinical trials evaluating the usefulness of 5-azacytidine in HL treatment are ongoing, including the phase II study of its application in combination with pembrolizumab (NCT05355051) (**Table 1**). Another promising DNMT inhibitor is decitabine. Application of camrelizumab along with decitabine results in longer progression-free survival compared with camrelizumab alone in patients with relapsed/refractory classical HL (85).

### Histone modifications and histone modifying enzymes

Modifications of histone proteins are key epigenetic mechanisms that control gene expression and chromatin structure, and are both introduced and removed by specific enzymes. Histone modifications control accessibility of genomic DNA present in chromatin. Such covalent modifications of histone tails as acetylation and phosphorylation have a positive impact on the gene expression, as they lead to the loosening of chromatin structure. By contrast, other modifications may play a repressive role due to restriction of access to the DNA in regulatory sequences for various transcription factors and other regulators. These histone modifications play an important role in cancer development and progression.

#### Histone acetyltransferases (HAT) and histone deacetylases (HDAC)

Histone acetyltransferases (HATs) are enzymes that introduce (write) histone acetylation by depositing the acetyl moiety on lysine residues of histone proteins. Such modifications lead to chromatin de-condensation, and subsequently promote gene expression. HATs are categorized into five families with 17 members (86). In HL, mutations of various types (missense, nonsense etc.) have been found in CBP/p300 HAT. Some of them were located in the acetyltransferase KAT domain (87). Other studies have indicated that HL cells are characterized by hyperacetylation of 211 genes, while 327 genes were hypoacetylated, when compared to normal B-cell line. Genes that were hyperacetylated in HL were classified as involved in apoptosis regulation and cell death, the Toll-like receptor pathway, and myeloid differentiation. Among genes hypoacetylated in HL, the B-cell characteristic genes were identified. Collectively, the altered acetylation on gene *loci* is consistent with the characteristic features of HL cells (88).

Histone deacetylases (HDACs) are enzymes which remove acetyl groups from histone tails, leading to an increased positive charge, therefore promoting histone-DNA interaction. As a consequence, the chromatin structure is more tightly compacted. Thus, their function is the opposite of HATs. HDACs are classified as erasers of histone modifications. They may also act on numerous non-histone proteins such as HSP90, HIF1, and p53 that govern a wide range of biological processes including cancer initiation and progression (89). The HDAC family is categorized into 4 classes, comprising 18 isoforms. In HL, HDACs are overexpressed. HDAC class I (HDAC1, 3) and HDAC class IV (HDAC11) were significantly overexpressed in HL. In contrast, HDAC2 was downregulated in HRS cells (90, 91). Higher expression of HDAC1 and 11 correlated with shorter overall survival of HL patients, with lower HDAC2 expression correlating with the pathological HL subtype (91, 92). So far, several attempts aimed at HL treatment using HDAC inhibitors (HDACi) have been described. The HDACi were tested in a single treatment, or in combination with other compounds (93, 94). Phase II clinical trials for several of them, including Vorinostat (95), Mocetinostat (96), Panobinostat (93), and Entinostat (97) have been completed and the published results show promising opportunities for using these HDACis in the HL treatment (**Table 1**).

#### Histone methyltransferases and demethylases

Another epigenetic modification with profound impact on gene expression is histone methylation, occurring on arginine and lysine residues present in histone tails. Such modifications are introduced by lysine methyltransferases (KMTs) or protein arginine methyltransferases (PRMTs), which are classified as epigenetic writers. They use S-adenosyl-L-methionine (SAM) as the methyl group donor. Up to three methyl groups may be transferred to the same lysine, and up to two methyl groups may be added on arginine. Histone methylation may positively or negatively impact the expression of target genes. Knowledge regarding the importance of histone methylation in HL development and progression is still very limited. However, there are emerging data indicating that HL cells are characterized by increased levels of histone 3 trimethylation at lysine 27 (H3K27me3), representing a repressive histone mark on the promoter regions of genes involved in the B-cell characteristics (*CD19, CD20, CD79b, BOB1, PU.1, SYK, LCK, TCL1A, BCMA, PAX5*). In contrast, no enrichment for H3K27me3 was found on promoter regions of *CCR7, TRAF1, SEMA4C, IL6* genes, which are known to be expressed at high levels in HL. Consistently, the presence of repressive H3K27me3 trimethylation on B-cell characteristic genes correlated with H3 hypoacetylation in HL cells (88).

HL is characterized by silencing of nuclear factor of activated T cell 1 (Nfatc1). Nftac1 silencing showed correlation with decreased histone H3 acetylation and H3K4 trimethylation, as well as reduced Sp1 factor binding. Increased Heterochromatin Protein 1 (HP1) binding to the *NFATC1* promoter was identified. The hypermethylation of the promoter region in HL strongly indicates that the epigenetic regulatory mechanisms may be impaired in this type of cancer (98). Furthermore, increased histone 3 lysine 9 (H3K9) methylation was observed in the promoter region of the *IgH locus* in L428 and L1236 HL cells, consequently leading to the loss of immunoglobulin expression (99).

Histone demethylation executed by the histone demethylases (erasers) may also play an important role in HL development. It has been reported that KDM6B (JMJD3), the H3K27me3 demethylase, is overexpressed in HL after Epstein-Barr virus (EBV) induction. Depletion of KDM6B in HL cells leads to the increased H3K27 trimethylation on genes differentially expressed in this type of cancer, however the restoration of H3K27me3 did not cause their downregulation (100). Overexpression of other histone demethylases, KDM4B demethylating H3K9me3 and H3K36me3 and KDM4D demethylating H3Kme2, was also confirmed in HL. The overexpression was related to poor DFS, collectively indicating an important role for histone demethylation in HL development (101). In summary, alterations of histone modifications in HL represent a valuable target for further study, with special focus on understanding their role in the control of genes characterized by altered expression in this type of cancer.

### ATP-dependent chromatin remodeling complexes (CRCs)

Chromatin remodeling complexes (CRCs) are a group of multisubunit complexes, which are evolutionarily conserved in eukaryotes. CRCs use energy derived from ATP hydrolysis, and act in various ways on nucleosomes. This results in their repositioning, ejection, or exchange, thus giving or restricting access for transcription factors to their target sequences in genomic DNA. CRCs therefore play an important role in the transcriptional control of gene expression (102, 103).

CRCs are classified into four subfamilies, based on the type of the central ATPase subunit. These include SWI/SNF, ISWI, NuRD/Mi-2/CHD1 and INO80. The core of human SWI/SNF is composed of a central ATPase (BRM or BRG1), two SWI3-type (BAF155 and BAF170), and one SNF5-type (hSNF5/INI1/BAF47) proteins, however non-canonical variants of such complexes reportedly also exist in the cell (104). Genetic alterations of particular SWI/SNF subunits are frequently reported to be involved in cancerogenesis (105–107). A recent study indicated that SWI/SNF is mutated in about 25% of various cancers (108). The truncating and splice site mutations in the *ARID1A* (non-core AT-rich interactive domain 1A) gene encoding the BAF250A subunit of SWI/SNF CRC were reported in about 26% of HL patient samples, analyzed by the whole-exome sequencing of flow cytometry–sorted HRS cells from 23 excisional biopsies. The *ARID1A* acts as a tumor suppressor in multiple solid tumors, and in certain hematologic malignancies such as follicular lymphoma. Of note, the HLs with a *ARID1A* mutation exhibited an increased number of driver events, compared to HL carrying wild-type *ARID1A* (109). However, the relationship between impairment of the SWI/SNF complex and tumor-related genes in HL remains elusive and requires further investigation.

In the case of NuRD CRCs, CHD1 core subunit downregulation was a result of promoter methylation (110). Furthermore, it has been shown that the CHD3 subunit interacts with CD30 (Ki-1/57) protein, acting as a lymphocyte co-stimulatory molecule (111, 112). This may suggest a role for the NuRD/Mi-2/CHD complex in HL pathogenesis. To date, the existence of mutations in other ATP-dependent CRCs (ISWI and INO80) in HL has not been reported. Given the lack of a broad picture of alterations of chromatin remodeling complexes in the development and progression of HL, further research in this direction may prove valuable, leading to the deciphering of novel mechanisms related to transcriptional control of gene expression affected in this type of cancer.

### Polycomb-group (PcG) complexes

PcGs represent an important group of proteins that regulate gene expression and chromatin state (113). Human PcG proteins are present in two different complexes. These are: PRC1 (consisting of BMI1, MEL18 and RING1 core subunits and other proteins), which is essential for the maintenance of gene repression; and PRC2 (core EZH, EED and YY1 and other auxiliary subunits), which seems to be involved in the repression of transcription and modulation of the stem cell pluripotency, differentiation, and cell proliferation (114). The PRC2 complex mostly acts antagonistically to SWI/SNF CRCs, however these two complexes cooperate in the case of some genes (115). SWI/SNF plays an important role in PRC2 eviction (116). PRCs are significant in the development of B-cells, and in germinal center formation (117, 118). Distinct steps of B cell development are associated with dynamic changes in PcG gene expression profiles. The equilibrium between PRCs is critical for hematopoietic cell division, and essential for lymphoid cell proliferation. This equilibrium involves the EED/EZH2 and BMI1/RING1 proteins. The first is a positive regulator of lymphoid differentiation, and the latter is a negative one. A balance between these two complexes is disrupted in HRS cells. During B cell development, only one PRC complex is active, whereas the expression of the other one is diminished. However, in HRS cells, both PRC complexes remain active (118, 119).

The cyclin dependent kinase inhibitor 2A (*Cdkn2a*) gene plays an important role in cell cycle regulation. It encodes, among others, two proteins known as p16 and p19, which are major inhibitors of cell cycle progression. Cdkn2a is one of the targets of BMI1. A lack of Bmi1 leads to down-regulation of p16 gene in transgenic mice, while 27 of 52 BMI1 positive cases of HL exhibited strong nuclear expression of p16 (120). PcG proteins, including BMI1, RING1B, and SUZ12, directly bind the *p16* locus, subsequently leading to repression of pRB. PRC2-dependent trimethylation of H3K27 at this locus requires an active pRB protein, indicating a regulatory feedback loop between p16 expression and pRB-PcG complexes (121). The genes encoding PRC2 subunits are under the direct control of protooncogene MYC, which is another example of an overexpressed protein in HL (122). This overexpression is connected with an active JAK/STAT pathway, and activity of the BATF3 protein (which belongs to the AP-1 family). The expression of the BATF3 gene is driven by STAT factors. Subsequently, BATF3, along with JUN, initiates the expression of *MYC* gene (123).

The other members of the PRC1 complex, such as RNF2, MEL-18, EED, YY1 or RYBP, are also overexpressed in HL. RYBP protein is a part of the PRC1 complex, which plays a role in cell development and apoptosis. It interacts specifically with RING1A and YY1 proteins, belonging to PRC1 and PRC2 complexes, respectively (124). RYBP is overexpressed in 55% of HL, but is absent in normal lymphoid tissue and the lymphocyte-predominant type of HL. Its expression is positively associated with unfavorable treatment response and poor overall survival (125). Several PcG proteins interact or co-localize with distinct non-PcG proteins, including factors like CtBP, E2F6, KyoT2, AF9, SSX. These oncogenic proteins are not only able to impact the silencing activity of PcG complexes, but also have the ability to bind chromatin (126). Hence, up-regulation of PRCs in HL not only disturbs normal progression of the B cell cycle, but also results in enhanced oncogenesis. EZH2, a functional enzymatic subunit of PRC2 was found to be overexpressed in HL cells (127). Targeting EZH2 for cancer therapy has recently become a valuable and attractive research topic. Different types of EZH2 inhibitors have been developed (128) with Tazemetostat being approved by FDA for routine use in epithelioid sarcoma treatment (129). Recently phase I clinical study of EZH2 inhibitor SHR2554 (**Table 1**) was conducted on patients with relapsed or refractory mature lymphoid neoplasms including HL and it has shown that the molecule was safe and exhibited promising antitumor activity (130).

### Non-coding RNAs (ncRNAs)

ncRNAs are RNA molecules transcribed from DNA, which are not translated into proteins. All three RNA polymerases can transcribe ncRNA from both coding and non-coding DNA regions. The ncRNAs are divided into 2 groups based on their size –small ncRNAs (sncRNAs), which are < 200 bp, and long ncRNAs (lncRNAs), which are >200 bp. sncRNAs are further classified into PIWI-interacting RNAs (piRNAs) or microRNAs (miRNAs), which differ in size and some other properties. ncRNAs can act as modulators in epigenetic or post-transcriptional gene regulation (PTGS). ncRNAs have emerged as both key diagnostic biomarkers and therapeutic targets in various malignancies (131).

#### Small non-coding RNAs (sncRNAs)

It has been shown that the PIWI regulatory pathway may be affected in HL (132). This pathway involves PIWI interacting RNAs (piRNAs) controlling transposable element mobilization, germline (133) and cell development (134). PIWI proteins including PIWIL1, PIWIL2, and PIWIL4 exhibited various expression levels among HL cell lines, and could be detected in HL tissues. piR-651, piR-20365 and piR-20582 were expressed at higher levels in HL lymph nodes than in reactive lymph nodes (RLN) and HL cell lines. It was shown that piR-651 was highly expressed in the cytoplasm of HRS cells. In addition, clinical data suggested that low levels of piR-651 correlated with shorter DFS, OS, and lack of response to first-line treatment (132).

More is known about the action of small non-coding RNAs, namely the miRNA class in HL, in relation to posttranscriptional gene silencing. miRNAs can bind either the 3′ UTR region or internal targeted mRNAs, and subsequently forms RNA-induced silencing complexes with Argonaute proteins. This leads to the degradation of the transcript or inhibition of protein translation (135). Regulation by miRNA is important in the biological processes of cell growth and differentiation. Tissue-specific aberrant expression has been linked to cancer development, hence the miRNA profile represents an attractive possibility for further exploration in relation to cancer diagnosis and treatment (136, 137). Since 2008, several studies have reported different miRNA profiles from HL cell lines, HRS cells, reactive lymph nodes, and GCB (138–141). Despite the different conditions and starting material, several miRNAs are observed consistently up- (let-7-f, mir-9, mir-21, mir-23a, mir-27a, mir-155, and mir-196a) or down-regulated (mir-138 and mir-150) in HL. The observed cohort of miRNAs was categorized based on their targets, including impairment of B cell development (mir-9, mir-155, and mir-150), NFκB hyperactivity (mir-155), and immune evasion (mir-9 and mir-138) (142). Recently, miRNAs (mir-339-3p, mir-148a-3p, mir-148a-5p, mir-193a-5p and mir-4488) were found to exhibit suppression caused by DNA hypermethylation (143),. mir-148a was found to target the proinflammatory cytokine *IL15* gene, and the genes *HOMER1*, *SERPINH1* and *SUB1*, which influence cell growth and survival. mir-148a also plays an important role in negative regulation of proliferation in the KM-H2 HL cell line (143).

#### Long non-coding RNAs (lncRNAs)

lncRNAs are defined as untranslated RNAs of more than 200 bp in length. They play a critical role in a broad spectrum of biological processes, such as maintenance of genome integrity, genomic imprinting, cell differentiation, and development. In cancer, lncRNAs are involved in regulation of gene expression, including through chromatin remodeling, transcriptional and post-transcriptional regulation, a variety of chromatin-based mechanisms, and *via* cross-talk with other RNA species. Metastasis-associated lung adenocarcinoma transcript 1 (MALAT1) is an evolutionarily conserved lncRNA with an 8.7 kb long transcript, encoded on chromosome 11q13. MALAT1 plays a critical role in the maintenance of the proliferation potential of early-stage hematopoietic cells (144) and is associated with proliferation and metastasis in several malignancies (145, 146). Overexpression of MALAT1 was investigated in acute myeloid leukemia (AML) patients and AML cell lines, and was found to promote cancer progression *via* mA RNA modification of *ZEB1* gene (147). In addition, MALAT1 expression was increased in AML patients with isocitrate dehydrogenase 2 (*IDH2*) mutations and in murine models of Ten Eleven Translocation 2 (*TET2*) deficiency. It has been also shown that overexpression of MALAT1 involves hematopoietic stem cell and progenitor cell expression with enhanced inflammation in AML (148). In the L-428 HL cell line, miR-9 interacts with MALAT1 *via* two binding sites. Significant *MALAT1* overexpression was shown to be the effect of miR-9 and AGO2 suppression. Moreover, it has been shown that the AGO2-dependent pathway is important for MALATI degradation in the nucleus (149). Another report indicated differential expression for 639 lncRNA probes (corresponding to 475 lncRNA *loci*) in HL cell lines, when compared to the control GC-B cell line. 74% of differentially expressed lncRNAs were downregulated in HL. Three lncRNAs (FLJ42351, LINC00116 and LINC00461) which are upregulated in HL were further analyzed with respect to their function. The inverse expression between LINC00461 and MEF2C (myocyte-specific enhancer factor 2C) suggests an additional potentially important feature of HL (150).

## Conclusions

### Consequences of molecular alterations in HL, including prospective future treatments

Such epigenetic modifications as DNA methylation, histone modifications, altered nucleosome positioning and action of non-coding RNA, influence the chromatin structure (**Table 2**). This enables precise control of compaction of DNA in the cell nucleus. Chromatin undergoes dynamic multilevel spatio-temporal reorganization, controlling access to the target sequences in DNA. Results from the chromosome conformation capture (CCC) method suggest the existence of domains in the structure of a chromosome. Genes within these domains preferentially interact with each other, and may be co-regulated (151, 152). These domains are also known as topologically associating domains (TADs). TADs might contribute to genome regulation through looping interactions, where an enhancer sequence regulates a particular target gene (151).

**Table 2:** Regulatory pathways aberrant in HL.

Epigenetic mechanisms		HL model	Outcome	References
DNA methylation		Cell lines, primary B cells, and biopsies	Hypermethylation of B-cell specific genes’ promoters	Ushmorov et al. (68), Guan et al. (69), Overbeck et al. (70), Yuki et al. (71), Bohle et al. (72), Watanabe et al. (73), Ammerpohl et al. (74), and Eberle et al (77)
Histone modifications				
Histone acetylation		Cell line	Mutations in KAT domain encoding region of *CBP/p300 HAT* gene	Barretina et al. (87)
		Cell lines	Histone H3 hypoacetylation of the B-cell specific genes promoters, and hyperacetylation of the genes involved in apoptosis, Toll-like receptor pathway and myeloid differentiation	Seitz et al. (88)
Histone deacetylation		Biopsies and clinical data	Overexpression of class I and IV HDACs and downregulation of HDAC 2	Adams et al. (90), and Huang et al. (91)
Histone methylation		Cell lines	Reduction of H3K27me3 on promoters of B-cell specific genes, reduction of H3K4me3 on *NFATC1* promoter, and elevation of H3K9 methylation on promoter region of the *IgH locus*	Seitz et al. (88), Akimzhanov et al. (98), Ushmorov et al. (99)
Histone demethylation		Primary B cells, biopsies, and clinical data	Overexpression of demethylases encoding genes including *KDM6B*, *KDM4B*, *KDM4D*	Anderton et al. (100), and Bur et al. (101)
Chromatin Remodeling Complexes (CRCs)				
SWI/SNF subunits		Biopsies	Mutation of *ARID1A* gene	Wienand et al. (109)
NuRD		Biopsies	Hypermethylation of *CHD1* gene promoter	Dhiab et al. (110)
PcG		Biopsies	Overexpression of PRC1 subunits’ encoding genes including BMI-1, EZH2, RNF2, MEL-18, EED, YY1, and RYBP	Raaphorst et al., and (118) Sanchez et al. (119)
ncRNAs				
**piRNA**		Cell lines, biopsies, and clinical data	Decreased level of piR-651 correlated with HL lack of complete response to first line treatment	Cordeiro et al. (132)
**miRNA**		Cell lines, HRS cells, reactive lymph nodes, and GCB	uUpregulation of let-7-f, mir-9, mir-21, mir-23a, mir-27a, mir-155, mir-196a, and downregulation of mir-138 and mir-150	Navarro et al. (138), Gibcus et al. (139), Van Vlierberghe et al. (140), Yuan et al. (141)Paczkowska et al. (143)
lncRNAs		Cell line	MALAT1 degradation dependent of miR-9 and AGO2 pathway	Leucci et al. (149)
		Cell lines	Upregulation of FLJ42351, LINC00116, LINC00461, Downregulation of LINC00461, MEF2C	Tayari et al. (150)

A genome-wide SNP study led to the identification of associations which were significant for HL at 3q28, 6q22.33, 6q23.3 and 10p14. Using the Hi-C method (an evolution of CCC), looping chromatin interactions at 3q28, 6q23.3, 10p14 and 16p13.13 regions were found. Subsequently, the biological relevance of these looping interactions was evaluated in HL development. These interactions involved BCL6 and mir-28 at 3q28, MYB and ALDH8A1 at 6q23.3. Regions interacting with TAF3 were found at 10p14. In addition, the region 16p13.13 was found to interact with RMI2 (153).

**Figure 3:**
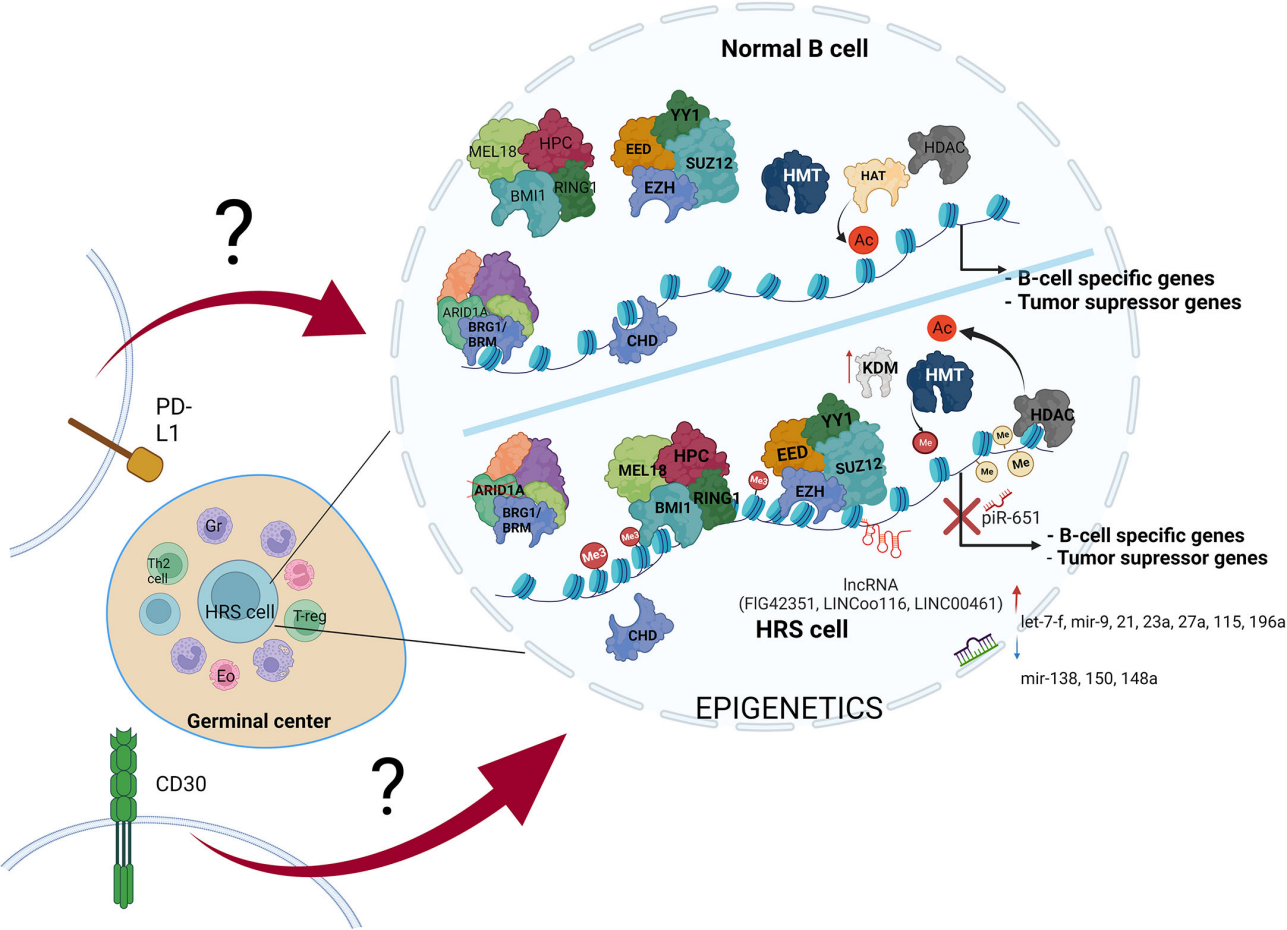
The variety of molecular alterations in HL. The main hallmark of HRS cells is overexpression of PD-L1 and CD30 molecules, and epigenetic machinery deregulation. The stimulation of transmembrane molecules activates signal transduction, but the influence of CD30 stimulation and PD-L1 reverse signaling on chromatin modifiers remains elusive.

Epigenetic alterations appear to be of critical importance for HL development (**Figure 3**, **Table 2**). Further exploration of epigenetic alterations for therapeutic purposes would therefore make sense. Feasible approaches could include drugs which target the role of chromatin/DNA modifiers, or epigenetic machineries, which are already used in treatment of diseases involving epigenetic abnormalities (154, 155). Another strategy could involve studying the influence of alterations in epigenetic machinery on particular processes and/or signaling pathways which are affected in HL. This could reveal additional novel mechanisms, which could represent attractive molecular targets for future HL treatments.

Alterations in B cell development result in inhibition of tumor suppressor genes expression, leading to transformation into HRS cells. These alterations are driven by multiple mechanisms, including such epigenetic alterations as DNA hypermethylation, enhanced histone deacetylation, histone hypermethylation, missing subunits of chromatin remodeling complexes-CRCs (SWI/SNF and CHD complex), PcG overexpression, increased expression of ncRNAs (piR-651, FLJ42351, LINC00116 and LINC00461), dynamic changes in miRNAs profile, and last but not least overexpression of PD-L1 and CD30 molecules (along with activation of their pathways in HRS cells) (**Table 1**). Therefore, studies aiming to couple the impairment of particular epigenetic machinery with specific regulatory process in HL would likely open new possibilities for development of novel therapies.

**Figure 4:**
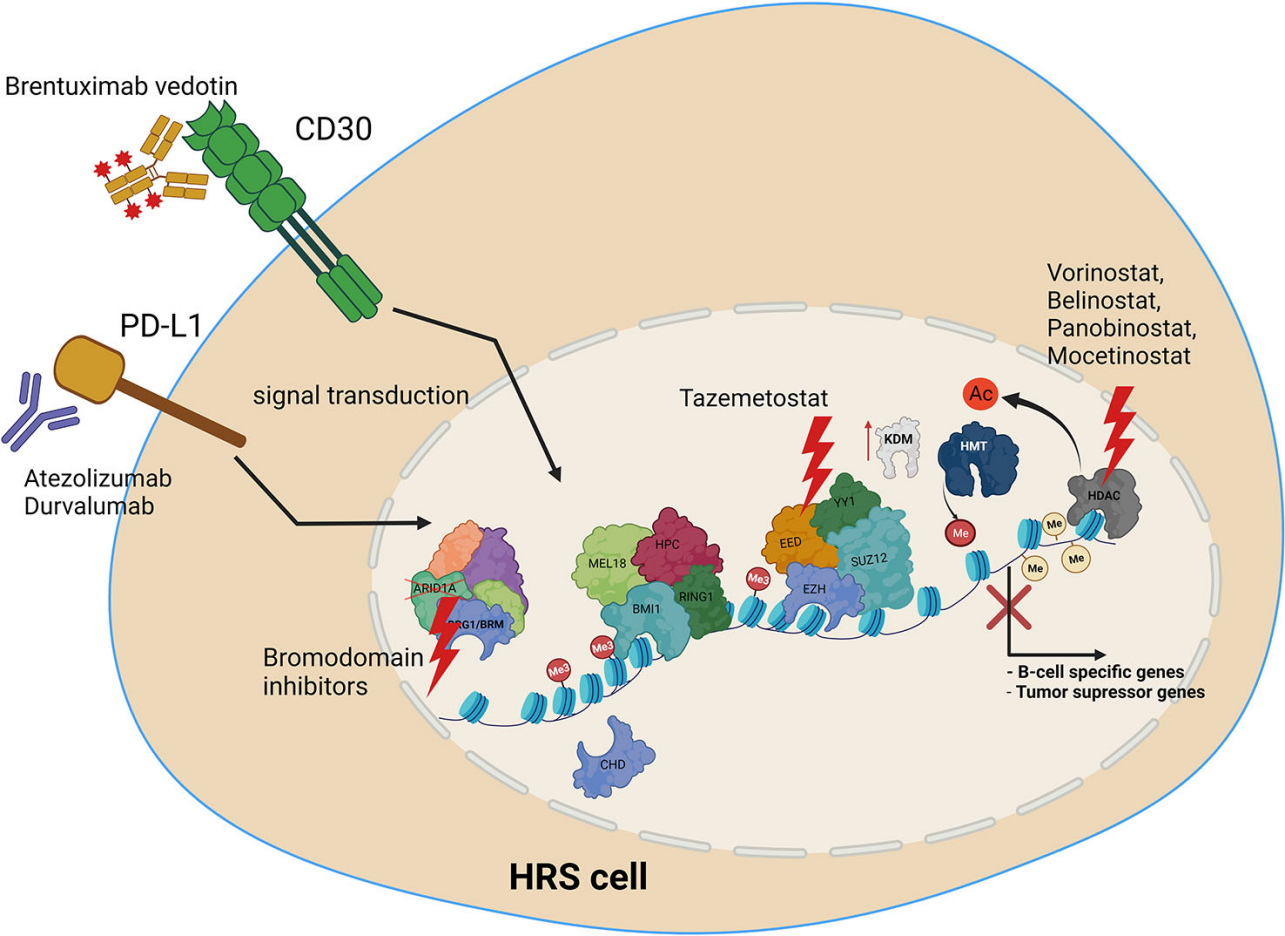
Molecular targets in HRS cells, along with potential uses of current and novel treatment options in combined therapy, to treat relapse and refractory HL. Treatment includes anti-PD-L1 or anti CD30 antibody, HDAC inhibitors, EZH2 (PRC2) as well as bromodomain inhibitors targeting SWI/SNF chromatin remodeling complexes.

Several treatments targeting epigenetic machinery have been recently tested for HL. These include HDAC inhibitors, such as Panobinostat, Mocetinostat or Belinostat (154, 156, 157). HDAC1 and HDAC2 are targeted by the Mocetinostat. Panobinostat has been tested both alone, and in combination with Everolimus, which is routinely used in the treatment of renal cancer (156, 157). Etinostat belongs to the benzamide drug class, and also targets HDAC. It appears to be a promising drug, retrieving the vulnerability of cancer cells to chemotherapy (156, 158). As recently tested novel treatments are mostly on histone deacetylases, it is vital to extend screening to include compounds with targets other than HDACs. We therefore propose the following open topics as a basis for further research, in order to identify potential novel molecular mechanisms for targeted HL therapy (**Figure 4**). Firstly, the potential use of EZH2 inhibitors, HDAC inhibitors, or BRD (bromodomain) inhibitors. Additionally, the use of anti-PD-L1 therapy, providing the opportunity to inhibit also PD-L1 reverse signaling. Furthermore, the use of combined anti-CD30 and anti-PD-L1 therapy, or treatments targeting epigenetic machinery together with anti-CD30 or anti-PD-L1. Finally, deciphering how PD-L1 reverse signaling and CD30 signaling may influence the chromatin remodeling, and *vice versa*. Chen and coworkers recently indicated the high efficacy of anti- PD-1 treatment after failure of anti- PD-L1 therapy in relapsed/refractory HL, although the patient group in this study was of limited size (35). Moreover, a multicenter phase 1-2 clinical study, iMATIX, revealed the limited efficacy of the anti-PD-L1 inhibitor atezolizumab in HL treatment (159). Study of this area therefore needs to be intensified, and alternative treatment options for refractory/relapse HL must be found.

## Author contributions

Writing—original draft preparation, HS, MA, RB, JK, JS, ES, TS, RK. Writing—review and editing, ES, TS, RK, SM, JW, EP-K. Supervision, TS, RK. All authors contributed to the article and approved the submitted version.
